# Perioperative Oxidative Stress and Sepsis: Pathophysiological and Clinical Implications

**DOI:** 10.7759/cureus.94719

**Published:** 2025-10-16

**Authors:** Alice Nicoleta Dragoescu, Vlad Padureanu, Andreea Doriana Stanculescu, Maria Andrei, Mihai Radu, Rodica Padureanu, Dominic Gabriel Iliescu, Petru Octavian Dragoescu

**Affiliations:** 1 Department of Anesthesiology and Intensive Care, University of Medicine and Pharmacy of Craiova, Craiova, ROU; 2 Department of Internal Medicine, University of Medicine and Pharmacy of Craiova, Craiova, ROU; 3 Department of Anesthesiology and Intensive Care, Emergency County Hospital Craiova, Craiova, ROU; 4 Department of Urology, University of Medicine and Pharmacy of Craiova, Craiova, ROU; 5 Department of Obstetrics and Gynecology, University of Medicine and Pharmacy of Craiova, Craiova, ROU; 6 Department of Urology, University of Medicine and Pharmacy of Craiova, Craiova, ROU

**Keywords:** antioxidant therapy, oxidative stress, pathophysiology, perioperative, sepsis

## Abstract

The relationship between oxidative stress and sepsis determines how well patients recover from surgery and critical illness. The perioperative period creates conditions for excessive reactive oxygen species (ROS) and reactive nitrogen species (RNS) production through surgical trauma and anesthesia and tissue hypoperfusion and infection, leading to mitochondrial damage, endothelial harm, and immune system problems. The processes create more inflammation while damaging cellular energy systems, which results in multiple organ system failure. The review examines modern knowledge about perioperative oxidative stress and sepsis mechanisms while discussing treatment implications and new therapeutic approaches. The authors conducted database searches in PubMed, Web of Science, and Scopus to find English-language peer-reviewed studies about oxidative stress and perioperative sepsis. The authors used the keywords “oxidative stress”, “perioperative”, and “sepsis” to find relevant studies from 2000 to 2025. The most effective strategy to reduce oxidative damage and enhance patient results involves early infection control, normoxia preservation, and accurate fluid management during perioperative care. The current understanding of oxidative stress has not led to the successful clinical implementation of targeted antioxidant treatments. Research should concentrate on developing individualized antioxidant treatments based on biomarker tests while conducting clinical trials to assess mitochondrial protective effects. The integration of mechanistic knowledge with clinical practice makes oxidative stress modulation a fundamental approach to enhance survival rates of patients with sepsis and those undergoing high-risk surgery.

## Introduction and background

The imbalance between reactive oxygen species (ROS) production and antioxidant defence systems results in macromolecular damage, which defines oxidative stress. The perioperative period triggers oxidative stress through multiple factors, which include surgical trauma, ischemia-reperfusion injury, high-concentration oxygen administration, and anesthetic agents [1,2].

Sepsis, as defined by the Sepsis-3 criteria, is a life-threatening organ dysfunction caused by a dysregulated host response to infection [3]. The development of sepsis depends heavily on oxidative stress because it functions as both an initial trigger and a resulting effect of systemic inflammation. The combination of oxidative stress with immune dysregulation in surgical patients who develop sepsis postoperatively leads to increased tissue damage and a higher risk of multiorgan failure [4].

This review aims to discuss the pathophysiological mechanisms by which perioperative oxidative stress predisposes to and exacerbates sepsis and to evaluate the clinical implications for monitoring and therapeutic intervention.

Although not conducted as a systematic review, this paper follows a structured narrative methodology to summarize current evidence in a clear way. The literature search used PubMed, Scopus, and Web of Science databases to find studies with the search terms "oxidative stress", "perioperative", and "sepsis". The research included studies from 2000 to 2025, which were published in English. The review included human studies and animal model research that examined perioperative oxidative stress and sepsis in peer-reviewed articles. The review excluded conference abstracts, non-original studies, and duplicate publications from its analysis. The assessment of each article included evaluations for methodological quality, relevance to the review question, and identification of potential bias sources through examination of study design, sample size, and conflict of interest disclosure. A brief methodological summary was created to guarantee evidence synthesis transparency and reproducibility.

## Review

Common pathophysiological mechanisms

The biological pathways that connect oxidative stress to sepsis form multiple overlapping mechanisms that primarily affect mitochondria, vascular endothelium, and immune system cells. The shared biological pathways between sepsis and surgical patients explain why sepsis development leads to organ failure and death.

Mitochondrial Dysfunction: Central Core of Oxidative Imbalance

The cell's energy production through oxidative phosphorylation occurs in mitochondria, while these organelles simultaneously produce reactive oxygen species (ROS) during normal and disease states. The electron transport chain complexes I and III suffer damage during sepsis and major surgical insults, which cause mitochondrial respiration to become uncoupled. The process results in superoxide anion (O₂⁻) formation through electron leakage [5,6].

The condition of cytopathic hypoxia shows particular importance in sepsis because it results from normal oxygen delivery but impaired cellular oxygen utilization, which leads to mitochondrial dysfunction [5].

The inflammatory mediators TNF-α and IL-1β cause damage to mitochondrial DNA while breaking down mitochondrial membrane potential (ΔΨm) and disrupting ATP production, which results in anaerobic metabolism and lactate production [7].

The release of cytochrome c from damaged mitochondria into the cytoplasm activates caspase-dependent apoptosis [4].

The activation of mitochondrial permeability transition pores (mPTP) by oxidative stress causes further membrane potential loss, which results in organelle swelling before leading to necrotic cell death [8].

The innate immune response becomes more severe because damaged mitochondria produce damage-associated molecular patterns (mtDAMPs), including mitochondrial DNA and formyl peptides, which act as potent inflammatory inducers [9].

Endothelial Injury and Microcirculatory Dysfunction

The vascular endothelium, which controls vascular tone and barrier function and hemostasis, shows high sensitivity to oxidative stress. The reaction between nitric oxide (NO) and superoxide produces peroxynitrite (ONOO⁻) directly damages endothelial cell membranes to increase permeability and cause capillary leak [10].

During sepsis, endothelial dysfunction causes impaired vasoreactivity together with leukocyte adhesion and microthrombosis, which results in uneven microcirculatory flow patterns and tissue hypoxia. The depletion of endothelial NO through ROS reactions intensifies the condition by causing paradoxical vascular tone increases and vasodilatory capacity loss in specific areas [11].

The glycocalyx layer, which protects the endothelial surface from damage, breaks down when exposed to oxidative conditions. The release of syndecan-1 from glycocalyx structures during sepsis and surgical procedures leads to poor patient outcomes because it enables uncontrolled leukocyte adhesion, intensifies inflammation, and causes capillary leakage [12].

The exposure of subendothelial tissue factor through endothelial damage triggers the coagulation cascade, which leads to disseminated intravascular coagulation (DIC) as a characteristic feature of severe sepsis [13].

Immune Dysregulation

The immune response in sepsis and surgery experiences significant modulation through oxidative stress mechanisms. ROS functions normally to help neutrophils and macrophages eliminate pathogens through the respiratory burst process. ROS function as second messengers that activate pro-inflammatory transcription factors NF-κB and activator protein-1 (AP-1) when present in excessive amounts [14].

NF-κB activation triggers the transcription of inflammatory mediators TNF-α, IL-6, IL-8, and iNOS, which produce elevated nitric oxide (NO) levels through their expression [15].

The NOD-like receptor protein 3 (NLRP3) inflammasome gets activated by ROS to produce mature IL-1β and induce pyroptosis, which represents an inflammatory form of programmed cell death [16].

The process creates a self-reinforcing cycle where inflammation generates ROS, which then enhances and prolongs the inflammatory response. The positive feedback mechanism produces systemic inflammatory response syndrome (SIRS), which commonly appears after surgery and during the beginning of sepsis [17].

The prolonged or excessive production of ROS results in immunoparalysis, which primarily affects antigen-presenting cells together with T-lymphocytes. The function of dendritic cells deteriorates while MHC class II expression decreases and T-cell apoptosis increases. The immunosuppressed state makes patients more vulnerable to additional infections, which leads to poor treatment outcomes [18].

Oxidative Injury to Cellular Mechanisms

The total impact of ROS affects all significant biomolecules in the body: (1) Lipid peroxidation damages membrane phospholipids, which results in cellular and organelle structural breakdown. The two main by-products of this process are malondialdehyde (MDA) and 4-hydroxynonenal (4-HNE), which serve as indicators of this process [19]. (2) Protein oxidation through carbonylation results in enzymatic dysfunction, which disrupts signalling pathways, receptor activity, and cytoskeletal structure [20]. (3) DNA damage that occurs through oxidation of mitochondrial and nuclear DNA (e.g., 8-hydroxy-2’-deoxyguanosine) leads to mutagenesis and apoptosis or cell cycle arrest [21].

The effects of ROS are not limited to immune cells because they occur in all tissues, including the heart, lungs, liver, kidneys, and brain, which leads to the multi-organ dysfunction syndrome (MODS) observed in septic and post-surgical critically ill patients [22].

Redox Imbalance and Reduction of Antioxidant Protection

The endogenous antioxidant systems, including glutathione (GSH), superoxide dismutase (SOD), catalase, and glutathione peroxidase (GPx), become overwhelmed and depleted under stress conditions. Research indicates that septic patients show significant decreases in plasma GSH and SOD levels, which directly relate to their illness severity and death rate [23,24].

Surgical patients who have poor nutrition along with diabetes or cancer, or chronic kidney or liver disease, show decreased antioxidant reserves, which increases their risk of oxidative damage during sepsis [24].

The concept of "redox vulnerability" emerged because susceptible hosts experience excessive tissue damage from minor oxidative stress [25].

Mitochondria-Immune-Endothelium Dysfunction

Recent research has established that mitochondria, together with immune cells and the endothelium, form a tightly connected system that causes organ failure during sepsis and perioperative oxidative stress [26].

The breakdown of one component leads to destructive effects on the other components: damaged mitochondria release DAMPs, activating immune cells; immune activation leads to cytokine storm and endothelial damage; and endothelial dysfunction promotes tissue hypoxia, worsening mitochondrial performance.

The interconnected nature of these components demonstrates why single-pathway treatments such as antibiotics and fluids may not be enough unless oxidative stress and inflammation are simultaneously managed.

Perioperative implications in septic patients

Major surgical procedures, including abdominal, cardiac, oncological, and transplant surgeries, trigger systemic inflammation and ROS production. The perioperative oxidative stress in patients who later develop sepsis leads to the following: acute lung injury becomes worse because of neutrophil activation and alveolar-capillary leak; the combination of ROS damage to tubular epithelial cells and endothelial dysfunction leads to worsening acute kidney injury; the myocardial contractility decreases and the vascular tone becomes less responsive to catecholamines; the condition leads to coagulopathy and DIC [22,27,28].

The risk of patients with comorbidities (e.g., diabetes, malnutrition, cancer, chronic pulmonary or renal disease) increases because their antioxidant defences are weakened and their bodies already have an unbalanced oxidative state [25].

Oxidative stress biomarkers in sepsis

Markers of Direct Oxidative Damage

Sepsis pathophysiology depends heavily on oxidative stress, which causes cellular damage through lipid and protein and nucleic acid destruction. The following biomarkers help evaluate the degree of oxidative damage: (1) MDA serves as a recognized indicator of lipid peroxidation because free radicals break down polyunsaturated fatty acids in cell membranes to produce MDA. The severity of systemic inflammation in sepsis directly correlates with elevated MDA levels, which indicate increased membrane damage. (2) 8-Hydroxy-2'-deoxyguanosine (8-OHdG) serves as a particular indicator of DNA damage caused by oxidation. The hydroxylation process of guanine residues produces 8-OHdG, which appears in urine or plasma measurements. The elevated levels of 8-OHdG indicate genomic instability due to oxidation, and these levels are commonly higher in septic patients, which may contribute to organ failure and negative treatment results [28]. (3) Protein carbonyls are formed by the oxidation of amino acid side chains in proteins, representing irreversible oxidative modifications. Systemic protein oxidation leads to elevated protein carbonyl content, which has been associated with negative prognostic outcomes in septic shock [20,29].

Markers of Antioxidant Defence Status 

The condition of sepsis leads to impaired antioxidant defence mechanisms, which occur simultaneously with increased oxidative damage. The following markers show the status of antioxidants [30-43]:

Reduced glutathione (GSH): The body uses GSH as its main antioxidant to detoxify ROS inside cells. The levels of GSH decrease substantially during septic shock, thus creating a redox imbalance, which makes the body more vulnerable to oxidative damage. Antioxidant enzymes: Superoxide dismutase (SOD) functions to transform superoxide anions into hydrogen peroxide and oxygen, which minimizes superoxide toxicity.

Catalase breaks down hydrogen peroxide into water and oxygen while working together with SOD to minimize oxidative stress.

Glutathione peroxidase (GPx) reduces hydrogen peroxide and lipid peroxides through the use of GSH as its substrate. The enzymatic antioxidants show decreased activity in patients with severe sepsis, which intensifies oxidative damage and leads to organ dysfunction [23,24].

The clinical use of these biomarkers remains limited, but research indicates their potential value in sepsis management. These biomarkers help doctors identify patients at high risk and track disease progression and assess the effectiveness of antioxidant-based treatments. The implementation of oxidative stress profiling in sepsis management would lead to better personalized treatment approaches, which would result in improved patient outcomes (Table 1).

**Table 1:** Oxidative stress biomarkers in sepsis Source: Refs. [39-43]

Variables	Biomarker	Type	Clinical Relevance	Prognostic Value
Markers of direct oxidative damage	Malondialdehyde (MDA)	Lipid peroxidation	Suggests membrane oxidative damage	High
	8-hydroxy-2'-deoxyguanosine (8-OHdG)	DNA oxidation	Suggests genomic oxidative stress	Moderate to High
	Protein Carbonyls	Protein oxidation	Suggests irreversible protein damage	Moderate
Markers of antioxidant defence status	Reduced Glutathione (GSH)	Antioxidant reserve	Suggests antioxidant depletion	Moderate
	Superoxide Dismutase (SOD)	Antioxidant enzymes	Suggests enzyme-based antioxidant defence	Variable
	Glutathione Peroxidase (GPx)	Antioxidant enzymes	Suggests enzyme-based antioxidant defence	Variable
	Catalase	Antioxidant enzymes	Suggests enzyme-based antioxidant defence	Variable

Therapeutic directions and interventions

Exogenous Antioxidants

Exogenous antioxidants play a potential therapeutic role in sepsis by modulating oxidative stress, which is a key contributor to cellular damage and organ dysfunction. Several agents have been investigated, either alone or in combination, with varying degrees of clinical evidence.

Intravenous vitamin C: The water-soluble antioxidant ascorbic acid functions as a strong vitamin C compound that neutralizes ROS and restores endothelial function. The compound shows anti-inflammatory properties during sepsis by blocking nuclear factor kappa B (NF-κB) activation and decreasing pro-inflammatory cytokine production. The compound helps create vasopressors, including norepinephrine, while enhancing blood flow through microvessels. The CITRIS-ALI trial studied high-dose intravenous vitamin C administration to patients who developed acute respiratory distress syndrome (ARDS) from sepsis. The study revealed decreased mortality rates and shorter ICU stays, but failed to achieve statistical significance for its main results, which continue to spark debate about its practical applications. The study revealed several promising secondary results that support additional research to explore its potential benefits [30,31].

N-acetylcysteine (NAC): The antioxidant glutathione (GSH) requires NAC as its precursor to function as an important intracellular antioxidant. NAC helps restore glutathione levels, which enables the body to maintain redox balance and detoxify ROS. NAC controls the activity of redox-sensitive transcription factors NF-κB and AP-1, which play a central role in sepsis inflammatory response. The established safety profile and strong mechanistic rationale of NAC do not justify its use in sepsis treatment beyond investigational purposes. The current body of research shows conflicting results about NAC's effect on patient outcomes, thus requiring additional large-scale randomized controlled trials to determine its effectiveness [32].

Selenium: It functions as a trace element that supports the antioxidant enzyme glutathione peroxidase (GPx) as its cofactor. The enzyme functions as a vital component to eliminate hydrogen peroxide and lipid peroxides. The condition of selenium deficiency affects critically ill patients who have sepsis and leads to elevated oxidative stress, impaired immune function, and negative treatment results. Clinical trials indicate that selenium supplementation could help decrease inflammatory markers and lower the risk of organ failure. Systematic reviews and meta-analyses have produced ambiguous findings about mortality benefits and clinical effectiveness, which has resulted in experimental status instead of standard practice for its use [33].

Mitochondrial Protection Strategies

The hallmark of sepsis includes mitochondrial dysfunction, which leads to multi-organ failure. The cell's powerhouses, known as mitochondria, generate ATP while controlling oxidative stress and apoptotic pathways. The cellular damage and energy collapse in sepsis result from ROS overproduction, mitochondrial membrane potential disruption, and electron transport chain dysfunction. Experimental and translational research now focuses on strategies that protect mitochondrial structure and operational capabilities.

Mitochondria-targeted antioxidants: The unique design of mitochondria-targeted antioxidants allows them to accumulate inside mitochondria, where they can neutralize ROS directly at their origin. MitoQ and SS-31 (elamipretide) represent two notable examples of such compounds.

(a) The lipophilic triphenylphosphonium cation in MitoQ enables this coenzyme Q10 derivative to penetrate mitochondrial membranes and reach high concentrations in the mitochondrial matrix. Preclinical studies demonstrate that MitoQ decreases oxidative damage while boosting mitochondrial respiration and decreasing inflammation during sepsis and other critical medical conditions.

(b) The small peptide SS-31 (elamipretide) targets cardiolipin, which exists exclusively in the inner mitochondrial membrane. The stabilization of mitochondrial membranes through SS-31 treatment leads to reduced cytochrome c release, which results in preserved mitochondrial structure and enhanced cellular energy production. The promising outcomes from animal research have not translated to clinical practice yet. The safety and effectiveness of these compounds are currently being studied through ongoing clinical trials for critically ill patients, but they have not been added to standard medical treatment protocols [34].

Ischemic preconditioning (IPC): The protective technique of IPC uses brief controlled ischemic episodes before long-lasting ischemia to improve tissue resistance. The protective approach demonstrates beneficial effects on liver, heart, and kidney tissues through its ability to control mitochondrial function and decrease oxidative stress levels. The protective mechanism operates through multiple pathways, which include enhanced mitochondrial antioxidant defenses and decreased ROS production during reperfusion and preserved integrity of the mitochondrial permeability transition pore. The implementation of IPC in liver and cardiovascular surgical procedures has resulted in better organ performance and decreased reperfusion damage. The experimental nature of IPC application in sepsis treatment remains the current state of research. The diverse nature of septic patients, together with the unpredictable patterns of systemic inflammation, creates obstacles for using IPC in critical care settings. The knowledge obtained from surgical models could lead to new strategies for protecting mitochondria during sepsis (Table 2) [35,44-47].

**Table 2:** Therapeutic antioxidants ROS - reactive oxygen species; GSH - glutathione; NF-κB - nuclear factor kappa B; GPx - glutathione peroxidase Source: Refs [30,39,44-47]

Agent	Mechanism	Clinical evidence	Limitations
Vitamin C	ROS scavenger Endothelial repair	CITRIS-ALI trial: positive secondary goals	Main goal not achieved
N-acetylcysteine	GSH precursor NF-κB inhibition	Variable results	Not standard care
Selenium	GPx cofactor Antioxidant support	Decreases mortality	Inconsistent meta-analyses results
MitoQ SS-31	Mitochondria-targeted antioxidants	Researches in animal models	Clinical efficacity not established

Perioperative Optimization

The optimization of perioperative care stands as an essential element for treating sepsis patients and those who might develop sepsis, especially in surgical environments. The implementation of specific interventions helps decrease oxidative stress while protecting tissues and maintaining stable blood pressure, which leads to better patient results. The following strategies represent key components of perioperative optimization.

Avoidance of prolonged hyperoxia [36]: The production of ROS increases when patients receive excessive oxygen through high inspired oxygen fraction (FiO₂) levels above 0.6, which results in tissue damage. Surgical patients, along with critically ill patients, require supplemental oxygen, but normoxic conditions, instead of hyperoxic conditions, help decrease oxidative stress and inflammation. The practice of limiting high FiO₂ exposure for extended periods represents a practical evidence-based approach to reduce oxygen-induced cellular damage during surgery [36].

Timely source control and optimized fluid resuscitation [48,49]: Sepsis management depends on immediate and successful source control, especially when the source is surgical, such as intra-abdominal infection, infected prosthesis, or necrotic tissue. The elimination of infection sources through surgical or interventional radiology procedures decreases both inflammatory signals and microbial loads. The management of organ perfusion requires individualized fluid therapy because excessive fluid administration leads to tissue edema and impaired oxygen delivery and increased risks of abdominal compartment syndrome and acute lung injury. The use of dynamic markers, including stroke volume variation and passive leg raise tests, enables healthcare providers to optimize cardiac output through targeted resuscitation strategies.

The following strategies help decrease surgical ischemia-reperfusion injury: The process of surgical procedures requires temporary blood flow interruption through vascular clamping and organ transplantation, which results in ischemia-reperfusion (I/R) injury after circulation restoration. The phenomenon generates significant oxidative stress and triggers inflammatory responses. Strategies to mitigate I/R injury include the following:

(a) The duration of clamp time should be minimized, particularly during major vessel and organ perfusion procedures, to decrease the time under ischemic conditions.

(b) The protective agents, which benefit endothelial and mitochondrial structures, should be used for pharmacologic preconditioning. The local anesthetic lidocaine shows protective effects against I/R-induced damage because it has anti-inflammatory properties and membrane-stabilizing effects [38].

The selective alpha-2 adrenergic agonist dexmedetomidine shows antioxidant and anti-inflammatory properties and organ-protective effects in both experimental and clinical research. The administration of dexmedetomidine during the perioperative period helps minimize I/R injury through its ability to control sympathetic tone and cellular oxidative responses (Table 3) [37,38].

**Table 3:** Perioperative optimization strategies ROS - reactive oxygen species Source: Refs. [48,49]

Intervention	Target	Evidence
Normoxia (FiO_2_ < 0.6)	Prevents ROS overproduction	Girardis et al, JAMA 2016
Early source control	Decreases inflammation Decreases infection	Sepsis-3 Surviving Sepsis Campaign (SSC) guidelines
Goal-directed fluid resuscitation	Ensuring adequate perfusion Prevents edema	ESPEN guidelines Surviving Sepsis Campaign (SSC) guidelines
Pharmacologic anaesthetic agents	Lidocaine Dexmedetomidine	Anti-inflammatory effects Antioxidant effects

## Conclusions

Despite significant advances in understanding the mechanisms linking oxidative stress and sepsis, effective targeted antioxidant therapies remain elusive. The most effective current approach for managing oxidative stress involves detailed perioperative care that includes immediate source control, normoxia preservation, and controlled fluid administration. Future research should focus on developing personalized antioxidant therapy based on biomarker profiling and conducting clinical trials of mitochondrial protectants to enhance outcomes in septic surgical patients.
